# The role of accurate identification of vulnerable youth in vocational education and training systems for improved employability: Insights from experimental data

**DOI:** 10.1016/j.dib.2023.109258

**Published:** 2023-05-22

**Authors:** Pallavi Gupta, Ambarish Datta

**Affiliations:** BSE Institute Ltd., 18th & 19th Floor, Phiroze Jeejeebhoy Towers, Dalal Street, Mumbai 400001, India

**Keywords:** Employability, Technical and vocational skills, Work-based scale, Behavioural scale, Interpersonal scale, Outcome evaluation

## Abstract

The role of vocational education and training system (VET) in addressing the economic and social needs of vulnerable youth from low-income households can be significant. It enables economic empowerment, providing them with a pathway to sustainable employment opportunities; improving their overall well-being and sense of personal identity. This article presents qualitative and quantitative data elucidating different elements of employability issues faced by such youth. It distinguishes and divulges a vulnerable group out of a larger population thereby making a strong case for identifying and addressing *their* needs. The approach is therefore not a ‘one size fits all’ training method. Students from urban Mumbai and New Delhi were mobilized from multiple channels such as self-help groups (SHGs), the National Institute of Open School (NIOS), distance education institutes, local government colleges, night schools and through direct community outreach. After carefully matching for demographic and economic characteristics, a group of 387 students in the age bracket of 18 to 24 years were selected and interviewed. This first set of data was generated for a range of personal, economic, and household characteristics. Data manifests structural barriers, poor human capital endowments and exclusion. To gain further insight into characteristics and to design a targeted intervention for a sub-group of 130 students from the total population, a second dataset is generated through a questionnaire and interview. Of this, two equal groups – an experiment group and a comparison group are formed as part of quasi-research. The third type of data is generated using a 5-point Likert scale questionnaire and personal discussions. A total of 2600 responses from the experiment (trained and skilled) and comparison (not trained) groups provides a basis for comparison of scores between the two groups pre-and post-intervention. The entire data collection process is practical, straightforward, and simple. Easy to explain - the dataset can be leveraged to generate evidence-based insights, and informed decisions on resource allocation, program design and strategies to mitigate risk factors. The multifaceted approach of data collection can be adapted to accurately identify vulnerable youth and create a newer framework for skill development and re-skilling. It can be used to develop measurement tools for employability by those involved in VET and in their efforts to create viable employment opportunities for high-potential yet disadvantaged youth.

**Specifications Table**

**Table:** 

Subject	Social Sciences/ Education
Specific subject area	Using a quasi-experimental research design to test the outcome of a skill development program on technical, interpersonal, and behavioural changes in experiment and control groups.
Type of data	Table Figures
How the data were acquired	432 students were mobilized from SHGs, night schools, government schools and NIOS. Data for demographic, personal, and educational characteristics were collected from personal interviews and online questionnaires in multiple stages of intervention. From this large database, a set of 130 closely matched students were selected and subsequently divided into experiment and control groups. Outcomes were tested post-intervention using a 5-point Likert scale questionnaire consisting of 20 questions, divided into 3 domains. Scores were assigned to create a weighted baseline index with a score range of 20 to 100 in each domain. SATA was utilized to analyze data.
Data format	Raw Analyzed
Description of data collection	The data collection process is conditioned to maintain the equality of two groups that are subject to quasi-experiment. Relevant economic and social variables to identify vulnerability patterns and their possible bearing on vocational training requirements are closely matched. Due to the small sample size, the Skewness-Kurtosis test for Normality is used to ensure data follows a normal distribution and to test the validity of the *t*-test.
Data source location	• Institution: Self-help groups (SHGs), direct community outreach at government schools, night schools, National Institute of Open School (NIOS), local colleges and distance education institutes in urban/ sub-urban Mumbai and New Delhi. • Training, successive data collection, running the quasi-experiment, outcome evaluation at BSE Institute Ltd., Mumbai, and New Delhi • City/Town/Region: Mumbai and New-Delhi • Country: India
Data accessibility	Repository name: Harvard Dataverse Data identification number: https://doi.org/10.7910/DVN/9QZL4E Direct URL to data: https://dataverse.harvard.edu/dataset.xhtml?persistentId=doi:10.7910/DVN/9QZL4E See Reference [1]
Related research article	Gupta, P., Datta, A., & Kothe, S. (2023). Developing Employability Skills in Vulnerable Youth: designing logic model framework and outcome evaluation using quasi-experiment. *World Development Sustainability, 2*(2023), 100045. https://doi.org/10.1016/j.wds.2023.100045 See Reference [2]

## Value of the Data

It is important to distinguish and bring the vulnerable group out of a general population and make a strong case to address *their* needs. The comprehensive dataset in this article includes relevant variables that can help identify vulnerable youth and design a customized intervention that includes flexibility, financial aid, mentorship, and counselling to help mitigate risk factors.

This data highlights not only obvious demographic and economic elements but specifically those that highlight risk factors common to low-income households in developing countries. Data shows that in vulnerable groups, more than developing technical skills, the returns to developing generic skills are higher [3,4].

Raw data can be used by those involved in skills development, employability activities or designing support strategies for vulnerable groups. They can leverage the dataset to conduct further statistical analysis such as regression or subgroup analysis or uncover patterns that may not have been apparent in quasi-experimental design alone.

There is a high potential for re-use of the data and transferability of the questionnaire to enable efforts towards inclusive and targeted vocational education and training systems across different countries. Questionnaire can be developed into a measurement tool to accurately identify at-risk students and areas for skills development.

This article also presents filtered and analyzed data for a selected group of students subject to a quasi-experiment. Data presents insight into some factors that have an impact on an individual's real chance of getting a job. If learning and employability are supportive constructs, this data reflects some factors that are responsible to cause unemployability and can thus help policymakers develop a better framework for skills development and training that suits their economic compulsions [5].

While the data focuses on vulnerable youth in the Indian context, shedding light on their demographic characteristics; the methodologies have wider applicability and relevance to explore similar issues and understand challenges faced by youth in low-income households across different regions.

## 1 Objective

There were three potential objectives for generating this dataset: First to accurately identify and classify vulnerable youth who are at risk of dropping out of school or college for reasons such as low household income, poor human capital endowment, social exclusion, or lack of protective factors. This involved collecting information across multidimensional personal, demographic, and educational characteristics. Data collection was designed to identify exclusion patterns and risk factors to distinguish and bring the vulnerable group out of the entire population and make a strong case to address their needs. Second, datasets were collected pre- and post-intervention as part of a quasi-experiment with the objective of testing program effectiveness in terms of developing not only work-based skills but simultaneously enhancing positive personal attributes that may effectively reduce their exposure to vulnerabilities. A third type of dataset was generated during the training program. This ongoing dataset was generated to identify patterns of skills development, or behavioural and interpersonal changes to help program modifications and improvements to ensure intervention is achieving its intended goal. The dataset provides a robust evidence base, lending credibility and validity to the published article. It enriches the original article by providing deeper insights into the extent of vulnerabilities and the impact of the skills development program.

## 2 Data Description

The data was generated from a micro-level online survey elicited through a questionnaire and converted into a Google form. Outreach helped mobilize 432 students that took the survey. Mobilization was from direct community outreach to government schools, and night-schools. National Institute of Open School (NIOS), local colleges and distance education institutes, and self-help groups (SHGs) in urban/ sub-urban Mumbai and New Delhi. Of the 432, 387 students came for pre-scheduled personal interviews.

The data from the survey is collected for demographic characteristics such as gender, location/ region, social class division, annual household income, type of accommodation/ household rented or owned, and ownership of assets such as television set/ two-wheeler or four-wheeler vehicle. Personal characteristics such as name, date of birth (to confirm age), education, gap years in schooling (if any) and reason, educational attainment of parents/ employment/ type of employment casual work/ full-time/ government employee/ unemployed. Refer to the file ‘**Template questionnaire skilling survey.docx**,’ in the repository [1], for the questionnaire. Data was also collected for students' perceived importance of training and skills development and their manner of future spending on financial support for family or investment in higher studies. The following points give a broad summary of the questionnaire:

• What is your age?
• What is your gender?
• What is your household annual income?
• How many people live in your household?
• How many earning members are in your household?
• Does your household own any of the following assets: TV, washing machine, computer/ laptop, or internet access, and type of vehicle owned?
• What type of house/ dwelling does your family have- owned or rented?
• What is your highest level of education completed?
• What is the highest level of education completed by your parents?
• Is there anyone in your family who has completed a bachelor's degree or higher?
• Are you a first-generation learner in your family?
• Are you currently employed? If yes, what is your current occupation?
• Have you completed any vocational training before?
• If yes, what kind of vocational training have you completed before?
• If no, do you think vocational training is important for your future?
• Have you ever faced any financial constraints that affected your education or employment opportunities?

Starting with those who are most likely to benefit, the responses can bring out elements of vulnerability faced by the youth from low-income households. These questions provide insights into the personal and educational characteristics of the respondents, as well as their household income and assets. The complete questionnaire is deposited in the repository [1].

Table 1 presents the summary of the total cleaned sample (n = 387) as a frequency table. This data is in the file ‘**Campus to Corporate_432.xlsx**’ in the repository [1]. The file consists of qualitative and quantitative data collected on characteristics mentioned previously. The data brings to light the disadvantages and vulnerabilities faced by these youth.

**Table 1:** Data summary (N = 387). (Adapted from Appendix 2, “Data Description”, in the original research article.

Category	Frequency	Per cent
Gender: Female	228	59%
Gender: Male	159	41%
Residence: Rural	108	27.90%
Residence: Urban	279	72.10%
Social class: General	279	72.10%
Social class: Scheduled Castes	57	14.80%
Social class: Other Backward Castes	45	11.50%
Social class: Scheduled Tribes	6	1.60%
Students with gap years	36	9.30%
Annual Household Income: <= Rs. 70,000	222	57.40%
Annual Household Income: Rs. 70,000 - 2,73,098	114	29.50%
Annual Household Income: > Rs. 2,73,098	0	0%
No. of earning members in the family: 1	234	60.70%
No. of earning members in the family: 2	89	23%
No. of earning members in the family: 3	38	9.80%
No. of earning members in the family >3	26	6.70%
Type of accommodation: Rented home	215	55.70%
Type of accommodation: Own home	172	44.30%
Household vehicle ownership: Yes	146	37.70%
Household vehicle ownership: No	241	62.30%
Students who perceive vocational training as crucial	302	78%
Students who are not sure	85	22%
Attained vocational training previously	133	34.40%
No employment after training	121	90.90%
Short run, one-time employment after training	12	9.10%
Students who want to utilize future salary as financial support for family and own education	387	100%

*Note:* The first column lists the demographic characteristics, the second column lists the corresponding number of the total, and the third column lists the percentage of students in that category.

Of 387 students interviewed, a group of 65 students were identified and they were the experiment group that was subject to quasi-experiment. A summary of the experiment group is presented in Table 2 Complete data describing the characteristics of this group are presented in the file **‘Campus to corporate placed.xlsx**,’ in the repository [1]. The general socioeconomic characteristics remain like that of the entire population except for higher male participants as compared to females (male = 51 and female = 14). This is the experimental group trained under the skills development program. Carefully matching with the experiment group, a similar comparison group (which is not subject to any type of training) is created [6,2]. The repository does not have separate data for the comparison group. Since the group is made from a larger population initially identified, and is similar in characteristics to the experiment group, this was not considered essential.

**Table 2:** Data summary (n = 65). (Adapted from Appendix 3, “Data Description of the experimental group”, in the original research article).

Category	Value	Count
Gender	Female	14
	Male	51
Residence	Rural	0
	Urban	65
Social classification*	General	47
	Scheduled Castes	7
	Other Backward Castes	11
	Schedules Tribes	0
Annual household income	<= Rs. 70,000	41
	Rs. 70,000 - Rs. 2,73,098	24
	> Rs. 2,73,098	0
No. of earning members in the family	One member	44
	Two members	21
	Three or more members	0
Type of accommodation	Rented home	30
	Own home	35
Household vehicle ownership	No vehicle	33
Students who perceive vocational training as crucial		65
Attained some form of vocational training previously		6
Short run, one-time employment after vocational training		5
Students who want to utilize future salary as financial support for family and own education		65
No. of dependent members in a household (>3)		0
Students with gap years		2

*Note:* The first column lists the category of data, the second column lists the corresponding value or category, and the third column lists the count or number of students in that category.

⁎ The Indian social classification based on caste or ‘caste system’ can help understand the vulnerabilities of youth belonging to lower social divisions or castes in India. The caste system in India is a hierarchical system that places people into different castes based on their birth. The lower castes, also known as the Scheduled Castes, have historically faced discrimination and marginalization. This discrimination has led to limited access to education, economic opportunities, and social mobility making it difficult for young people from lower castes to access education, vocational training, and other opportunities that would help them build their skills and improve their economic prospects [7]. Mobilization, however, could not reach out to a large portion of youth from the ‘lower caste’ stratum, and their participation remained low.

The Skills Development and Training Program is summarized in Table 3 It presents all important elements such as objectives and outcomes, method of delivery and type of modules for training, place and methods for mobilization, these elements were significant in making the program. Before the intervention, a baseline survey was conducted for the experimental (65 students) and comparison (65 students) groups. The survey consisted of questions designed considering factors that influence employability and skills development in the BFSI sector.

**Table 3:** Summary of skills development program. (Adapted from Appendix 1, “Summary of Key Features” in the original article).

Objective	Designing a targeted and sustainable employability framework within the vocational education and training system (VET) for vulnerable or at-risk youth.
Place of Implementation	Mumbai, New-Delhi
Implementing Agency	BSE Institute Limited, Mumbai
Method of mobilization	Outreach principals and teachers of such institutions to identify desired students and advertise skills development programs for increased mobilization. Identification of targeted groups through surveys, online questionnaires, and personal interviews.
Target Group	12th Pass or college Graduates from low-income or lower-middle-income households or likely school dropouts or at-risk yet high potential students likely to have faced social exclusion and economic vulnerabilities.
Target population age	18-25 years
Mode of delivery	Classroom learning/ online discussions/ in-class learning and evaluations/ on-site training.
Modules	Sequential, industry-driven, and job-oriented curriculum/group discussions/ presentations/ personal and group mentoring sessions.
Mobilisation	Multiple channels such as SHGs, Direct community outreach at night schools and distance education institutes, local government schools and colleges.
Outcomes	Developed in-demand and required work-based skills in two BFSI sectors- Microfinance Associate and Junior Data Analysis Associate. Visible behavioural and interpersonal skills. Improved motivation and a sense of self-worth.

*Note:* The first column lists the important elements of the skills development program, and the second column lists the explanation of that element.

Alongside learning sector-specific skills, the significance of building certain transferable skills or generic skills improves learning outcomes [8]. Creating self-efficacy or a sense of self-worth remains one of the most crucial elements of any training program and its significance for vulnerable youth cannot be over-emphasized [9]. Based on this understanding three domains were created to test skills pre- and post-intervention among the two groups. Table 4 below summarises the question within each of the three domains. Three broad parameters of testing skills within that domain are described as follows:

• **Domain A or work-based skills:** 1) Applied knowledge 2) Technological skills, 3) Information use.
• **Domain B or interpersonal skills:** 1) Effective communication skills 2) Personal presentation, 3) Time management.
• **Domain C or behavioural skills:** 1) Motivation and confidence 2) Future identity 3) Willingness, responsibility, and self-discipline.

**Table 4:** Summary of questions with each domain.

**Domain A**	
Q1	How confident do you feel in using technology relevant to your field?
Q2	To what extent do you feel equipped with the necessary job role skills?
Q3	How comfortable are you with the workplace skills and cognitive abilities necessary for your chosen career path?
Q4	How well do you rate yourself in terms of employability attributes such as communication, teamwork, and adaptability?
Q5	How satisfied are you with the academic assistance and support provided during your training program?
Q6	To what extent do you feel ready and prepared for job placement in your desired field?
Q7	How satisfied are you with the quality of your current job, if employed?
Q8	How confident are you in your literacy and numeracy skills?
Q9	To what extent are you able to effectively utilize information to succeed in your career?
**Domain B**	
Q1	Are you comfortable with communicating your ideas to others?
Q2	How often do you analyse and evaluate different perspectives on a problem before coming up with a solution?
Q3	How well do you manage your time and resources to meet your goals?
Q4	What transferable skills have you gained through your previous experiences that you can apply to your current work?
Q5	How do you prioritize and manage your tasks to meet deadlines?
Q6	Do you present yourself professionally in different settings, such as at work or in interviews?
**Domain C**	
Q1	How motivated are you to achieve your career goals?
Q2	How do you envision your future professional identity?
Q3	Are you open and willing to learn new skills and knowledge?
Q4	Do you take responsibility for your actions and decisions in the workplace?
Q5	How do you practice self-discipline to meet work-related deadlines and goals?

*Note:* The three Domains: A, B and C, mention questions that can measure the outcome in each domain. All questions are presented on a 5-point Likert scale. Domain A has 9 questions that create a score in a range of 1 to 45. Domain B has 6 questions creating a score within a range of 6 to 30 and Domain C has 5 questions, creating a score within a range of 5 and 25. Thus a total score of all three domains is created with a minimum value of 20 and a maximum value of 100.

To measure employability skills in students of experimental and comparison groups before the intervention, a 5-point Likert scale questionnaire was drafted and administered. The questionnaire consisted of a total of 20 questions: nine in Domain A, six in Domain B, and five in Domain C. Scores were assigned to each question to create a baseline index in the following manner: 5 = “strongly agree/ always/ excellent”; 4 = “somewhat agree/ often/ good”; 3 = “neither agree nor disagree/ neutral/ average”; 2 = disagree/ rarely/ poor”; and 1 = “strongly disagree/ never/ very poor”. Weights were assigned separately to each domain, such that a minimum score of 20 and a maximum score of 100 is created.

File ‘**BSE data files_quasiEx.xlsx**’ in the repository presents the scores generated using the 5-point Likert scale. Such scores are presented under the three domains for both experiment and comparison groups. A total of 2600 scores are generated for both the experiment and comparison groups before intervention and a total of 2420 scores are generated for both the groups post-intervention. Average scores, mean scores, and standard deviations of each domain are also mentioned. There are seven sheets in this file. Sheet 1 consists of perception data administered to students and employers. To identify employability skill perception amongst the experimental group, from a list of seven skills the experiment group is asked to rank these on a scale of 1 to 7. Rank 1 to the skill they perceive as most important and rank 7 to one considered least important. These skills are derived from 14 key employability skills as discussed in [10]. A similar skills perception survey is administered to 19 individual employers identified in the BFSI sector. Sheet 1 also gives the means and standard deviation of these skills. Sheets 2 and 3 have baseline survey or pre-intervention scores for all three domains for both groups. Sheets 4 and 5 have post-intervention survey scores for all three domains for both groups. Sheets 6 and 7 have combined data from all previous individual sheets.

It should be noted that post-intervention the number of students in the experiment group remained the same (n = 65) while 57 students from the comparison group remained post-intervention. Eight students did not respond to the questionnaire or were not available for calls.

## 3 Experimental Design, Materials and Methods

Data was collected through a survey administered to 432 students in Mumbai and New Delhi from government schools, night schools, and distance education institutes, to identify vulnerable youth from lower income households. From the 387 mobilized students, a comparison group and an experimental group were formed. Mean age is 21 years and around 40% are first-generation learners. Participants were selected based on academic performance, household income, demographic backgrounds, and commitment towards the program, using the same set of selection criteria and interviewers to control for selection bias and student variability.

Participants were tested based on four broad parameters:

1) Academic performance and orientation,
2) Household income,
3) Demographic backgrounds, and
4) Commitment for participating in the program.

The study conducted a quasi-experimental research exercise for five months with 65 students in the experimental group and 65 students in the comparison group. These groups were selected on basis of academic performance, household income, demographic backgrounds, and commitment towards the program. A fully refunded deposit fee of Rs. 5000 was taken to ensure commitment from the participants. Data was collected through quantitative and qualitative methods at three levels: baseline survey, assessment, and evaluation.

The baseline survey included pre-tests on factors that influence employability and skills development. Developing both sector-specific and generic skills or transferable skills is important [11,8], and more so for vulnerable groups [12]. Level 1 or ‘Baseline survey’ was used to select experimental and comparison groups, identify skill gaps, and design the curriculum. Level 2 or ‘Assessments’ involved a mixed-methods approach including standardized tests, class presentations, and computer-based assessments. All assessments were credit based and a composite credit was generated for each student based on their performance throughout the training program. The assessment did not require a pre-test and post-test design since the students were previously not trained for sector-specific skills. Level 3 or ‘Outcome evaluation’ focused on testing pre-determined parameters evaluating work-based, interpersonal, and behavioural skills. The parameters for each domain are discussed in the ‘Data description’ section, and a questionnaire administered in Level 1 ‘Baseline survey’ is deposited in the data repository [1].

**Consent Form**

Title: Demographic Data Collection from Students

**Introduction**: We would like to collect demographic information from you as part of the skills development and training program. This information will be used to better understand the needs and characteristics of students. We will ensure that your information remains confidential and is used only for identifying students for the program.

**Consent**: By agreeing to participate in this research study, you are giving your consent to share your demographic information with us. The information collected may include your age, gender, ethnicity, educational background, family income, and other relevant details. Your participation is voluntary, and you may choose not to answer any question that you are uncomfortable with. You may also choose to withdraw from the study at any time, without any penalty or consequences.

**Confidentiality**: We will ensure that your information remains confidential and is not shared with anyone else outside of the research team. All data will be stored securely and will be accessible only to authorized members of the research team. The results of this research will be reported in aggregate form and will not reveal your identity.

**Contact Information**: If you have any questions or concerns about this research study, you can contact [researcher name and contact information]. If you have any questions or concerns about your rights as a participant in this study, you can contact [institution name and contact information].

I have read and understood the above information and agree to participate in this research study.

Participant's Name: _____________________________

Participant's Signature: __________________________

Date: ________________

## Ethics Statements

The authors confirm that the relevant informed consent was obtained from all the students participating in the online survey and in-person interviews. Where the dataset is shared, personal participant data is completely anonymized. No data was collected through any social media platform.

## CRediT authorship contribution statement

**Pallavi Gupta:** Conceptualization, Methodology, Software, Data curation, Writing – original draft, Writing – review & editing. **Ambarish Datta:** Visualization, Supervision, Data curation, Validation, Project administration, Methodology.

## Declaration of Competing Interest

The authors declare that they have no known competing financial interests or personal relationships that could have appeared to influence the work reported in this paper.

## Data Availability

Skills development and training of vulnerable youth from low-income households using quasi experimental research (Original data) (Dataverse).
